# Case of Hypertrophy of the Heart with Valvular Disease

**Published:** 1850-01

**Authors:** Samuel Thompson

**Affiliations:** Albion, Ill.


### THE NORTH-WESTERN
### MEDICAL AND SURGICAL JOURNAL.

VOL. II.] JANUARY, 1850. [No. 5.

### Part I.—Original Communications.

### ARTICLE I.

*Case of Hypertrophy of the Heart, with Valvular Disease.*—By SAMUEL THOMPSON, M.D., of Albion, Ill.

On 11th June, 1846, J. B. negro, aged about 60, called at my office, complaining he was troubled with soreness of breast and constipation. In answer to enquiry as to the character of his stools, he described them as having been for some time past, "white." From the feel of the pulse, the expression of the countenance, &c., I told him I considered he had a disease of the heart. I was very busy at the time, and directed him to call again, giving him some active cathartics, combined with mercury, to remove the constipation, which was of course aggravating every symptom, and to act on the liver, evidently deranged, as demonstrated by the color of the alvine discharges.

On the 27th June, I saw him at his house. His shortness of breath, upon making any exertion, with dizziness, and distension of the frontal veins, had rapidly increased, with, in addition thereto, an inability to lie down. The pulsations of the heart were rapid, (I did not count them by the watch,)

but so equal were the first and second sounds of the heart, that it conveyed the impression to the ear of beating double at each impulse, and imparted to the hand, applied over the chest, a strong thrilling feeling. The pulse was synchronous with the cardiac impulse, but *comparatively* very feeble. The impulse of the heart could be felt over the greater part of the thorax, and seen distinctly through all his clothes, even when at some distance.

Below the epigastrium, commencing even in the left hypochondrium, was a distinct and rather prominent tumor, extending from the inferior margins of the true ribs, about six inches downwards, and reaching across into the right hypochondrium. This tumor was extremely tender to the touch. He dared not eat or drink, except in the smallest quantities, from the fear of a pain of a darting, paroxysmal character, commencing on the left side and running round to the right, and apparently passing through this tumor, which always followed any deviation from this cautious abstinence.

The bowels were purged, but there was no amount of feculent matter discharged; the stools being composed of yellow, slimy water. The urine was scanty and very red. The tongue was pale and very broad. His legs and feet were anasarcous, partly, doubtless, from his inability to lie down; and when the pain was great, he was obliged to lean on the tumor to obtain any ease. The diagnosis I formed was Hypertrophy of the Heart, with obstruction of the mitral valves, and regurgitation as a consequence; thence obstruction to the return of blood from the lungs, and of course obstruction to the contractions of the right ventricle, and the portal and caval circulation.

The character of the abdominal tumor, and its tenderness on touch, yet relieved by *pressure*, appeared more in character with colicky distension of the transverse colon: at the same time, neither its feel, nor the sound on percussion, as far as he would submit to such, warranted that opinion; while the circumstance of the long continued constipation to which he said he had been subject, made it just possible that it might arise from *fecal* accumulation in the transverse colon.

I put him upon pills of Blue Mass and Extract of Conium, with a combination of Digitalis, Squill, and Cream of Tart., as palliative means only, feeling quite sure that the disease was far too much advanced for any chance of cure. It had evidently, from the account of himself and family, been growing for years, succeeding to several neglected attacks of Rheumatism.

On the 2d July I saw him again, when my worst fears were fully confirmed; and I then cautioned his family never to leave him alone, as his death might occur at any time without a minute's warning.

On the 14th he died suddenly in his chair, while seated under a peach tree at his cabin door. I had strongly urged upon the family, in the event of his death, to allow me to open his body; and aided by their curiosity, and that of their neighbors, to understand the cause of such, to them, strange symptoms, I obtained a partial permission: and having bribed a little boy to let me know as soon as he was dead, I received immediate notice, and went down the next morning with my then student, now Dr. Hugh Ronalds, and about 12 hours after death made a post mortem examination, of which the following were my notes. It will perhaps be remarked that I ought to have bled him. I thought so then and afterwards, but he was very low and unwilling:

*Autopsy.*—The surface of the body was hardly cold---the internal parts were warm. The vessels of the skin were much engorged with fluid blood; so that the flow from several veins, after dividing the integuments of the chest, was quite troublesome. The liver was of great size, apparently normal in structure, and full of blood. The left lobe was rather firmer than usual. The right one was attached to the side and diaphragm by nearly its whole superior surface. The

Lobulus Spigelii was of the size of a large duck egg. The gall bladder was much thickened, its parietes being about ¼ of an inch through, and containing about ℥ ij of thick, dark reddish brown bile. The stomach appeared healthy, but the coronary veins were distended with blood. The Duodenum was thickened and felt granular on its mucous surface; but there were no appearances of recent inflammation. *The Colon was much contracted*, and the small intestines were injected and of a pink color. The Omentum was nearly destitute of fat, but the mesenteric glands were very large, and hanging in some places by distinct footstalks. All the veins of the face were injected. The Spleen was very large and firm.

In the Thorax, the left lung was healthy, but pale; with some slight hypostatic engorgement on the posterior side. The right lung was also pale, with some few adhesive bands, of old date, between the costal and pulmonary pleuræ. On the anterior edge of the lung were some few emphysematous bladders, about the size of kidney beans. Both the thorax and abdomen were full of yellow serum, and the pericardium contained about ℥ ij., or ℥ iij. of fluid. To the pressure of the effused fluid I attributed the pale color of the lungs. The heart was very large, and weighed without the pericardium, (but with the dilated pouch of Aorta about two inches long attached) after it had been washed clean in water, then immersed in alcohol, and held up to drain before weighing, one pound six ounces avoirdupois. The right ventricle was rather large and thin---its walls being in many places not more than two lines thick. There were clear white fibrinous concretions interwoven with the chordæ tendineæ, and also some clots of blood between the fleshy columns. The tricuspid valves were normal, as also those of the pulmonary arteries. The left ventricle and auricle, the aorta, and the rest of the large vessels, were full of blood. The coronary veins were full of fluid blood, and so much distended as to equal in size a man's little finger. The muscular pillars were of enormous size and strength. The mitral valves were entire, but greatly thickened and very white, while the free edges felt cartilaginous. The semilunar valves of the aorta were much diseased; one having its free edge pretty much in a normal condition. One was much ossified. The other was converted into solid, ragged cartilage, with a sort of fringed edge, and with bony, pea-like depositions beneath, and also united to its inferior edge. The osseous or cartilaginous matter, for it was difficult to decide to which it most truly belonged, appeared to be deposited within the true structure of the valves, and thus bulging out the membrane and changing their normal size and form. The depositions extended even below the origin of the valves, but the membrane was not, as I could see, abraded. The greatest amount of disease appeared to be where the superior angles of the valves approximate. They were at that place pretty much fused together, and so concontracted the opening into the aorta, that the point of my little finger would not begin to pass up through the orifice; and when the canal of the aorta was laid open above, it presented a conical, and almost crater-like opening of about ⅙ of an inch in diameter. The aorta above was very much dilated and very thin. The walls of the left ventricle were, at their thickest parts, about one inch through, and on the pericardial surface of the left ventricle, were some small fibrinous bands of old date, (being fully organized) uniting it to the pericardium. There was also a small patch of the same kind on the right ventricle.

Here was a case, clear in the distinctive marks of the principal, and what may be called the primary lesion: for unless we look upon the rheumatic attacks of former years, as the primary error, I think all other derangements of either structure or fusion, may be clearly traced to the cardiac disease. The hepatic hypertrophy—for engorged as the liver was, it was certainly, also, I believe, increased in actual substance—the hypertrophy, then I say, must surely be recognised as the result of an undue mass of blood retained in its vessels. But taking this to be its cause, how shall we describe it as hypertrophy which implies excess of nutrition, unless we are prepared to allow that venous blood in its transit to the heart can supply nutrition, and that the veins are capable of secreting like arteries? Yet we know that in one sense the portal veins do secrete. That the hypertrophy or increased growth could not have been the result of excessive arterial supply, is evident from the obstruction of the aortic orifice: and though it is fair to suppose, that by its increased efforts, the left ventricle succeeded for some time in distributing over the body, the whole of the blood returned to it by the pulmonary veins, still it was evident, (and is, I believe, in such cases generally evident to the finger on the pulse,) that the arterial circulation was embarrassed.

I make these remarks without designing to base any theory; but as reflections occurring to my own mind from the facts herein presented: for either the excess of nutrition must have been supplied from the blood of the portal circle, or by virtue of a most exalted condition of the powers of assimilation, have been derived, from a certainly not *more* than common supply of arterial blood; or else, as Andral suggests, from deficient activity in the absorbents, (the deassimilating powers) have been dependent on *their* failure to carry off those materials it is their function to remove; and thence an accumulation took place. But if we admit this as a cause of the hypertrophy, we need borrow a term from Dr. Barlow's Treatise upon Plethora, and designate it as "Excrementitious" Hypertrophy, as of course the effete and exhausted molecules are all the absorbents, (be they veins or lymphatics,) ought to remove: and of such only, as result of their failure in function, could the redundancy be composed. The whole, however, appears pretty much beyond the grasp of our present knowledge, for as Dr. Townsend well remarks, "If we cannot comprehend how muscle or bone are formed from the blood in their due proportion, it need be no matter of surprise, if we cannot explain how these substances are occasionally formed in excessive quantities." I would suggest, however, whether the known fact that veins in a state of plethora absorb but slowly, if at all, while depletion, by lessening their contents raises their absorbing power to the highest grade, may not possibly furnish us some ideas upon the subject; adopting of necessity as therefrom resulting, Andral's suggestion, and calling to our own aid the observations of Dutrochet. We may perhaps yet discover that in venous tubes, in a state of great congestion, exosmotic may take the place of endosmotic currents, as in fact we see occur in dropsical effusions from obstructed venous circulation in the extremities, &c., and bearing in mind the peculiar character of the blood of the portal circle, loaded with newly ingested aliment, we may perhaps allow that a nutritive effect, though of an abnormal character, may result. The peculiar mode of distribution of the portal veins, assuming the arterial character of subdivision instead of convergence, in their course towards the heart, may also have an important bearing. For *why* arteries should secrete and veins absorb, we do not know. We know it is a fact, and we assume that it depends on peculiar nervous influences; but may it not possibly in some measure at least, depend on the subdivision they undergo; increasing their area, and thereby slackening their currents? whereas in veins, in general, the area decreases as the size of the trunks increases, and the velocity of the current is accelerated thereby. If there is any thing in this as a cause of arterial secretion, the same will hold good in reference to the portal veins; and we do know, that peculiar as they are in their mode of distribution, they are also peculiar in secreting, viz: the bile. All this is theorizing, I admit; but still, on some matters, till better informed, reasoning on facts we know, and thence deducing explanations of what we do not understand, is certainly allowable, and I think philosophical; Dr. Bartlett to the contrary notwithstanding.

That the hypertrophy of the *heart* must also have arisen in spite of obstructed circulation in the coronary arteries, and *without* any peculiarity in its venous blood or venous subdivisions, has to be borne in mind in reasoning npon this matter: at the same time I would remark, that the enormously increased muscular efforts of the heart in the disease above described, may possibly have enabled it to appropriate nourishment more rapidly from a common supply of blood. But to return to the record. The injected condition of the duodenum, the serum in the thorax and abdomen, are all clearly traceable to the impossibility of the left ventricle emptying its contents through the diminished orifice of the aorta, and its consequent inability to receive the supply sent by the right ventricle through the pulmonic system; hence the right ventricle became unable to propel its charge, and in its labored efforts so to do, became dilated, and also ruptured the pulmonary vesicles, producing the emphysematous blisters described, while the whole of the portal circle was in like manner dammed back, and the circulation passing by the superior cava was from the same cause obstructed, as was manifest in the distension of the frontal veins, and possibly was the cause of his sudden death, by pressure on the brain. (The head was not examined.) The case was remarkable as showing how the constitution seems able to accommodate itself to such important injuries, up to a certain point; for this man had, till about a month before his death, gone about his business, and only twelve months before, was chopping fire-wood for me, though at that time I remember he told me "his breath often failed him now." It was remarkable also, as an instance in a rather spare man, of a heart of nearly three times its normal weight, It was remarkable, I think, because that while defective action in the mitral valves alone would have given rise to regurgitation into the auricle, so great an obliteration of the aortic orifice inevitably doubled that effect, and thus of course produced not hypertrophy alone, but hypertrophy with dilatation; the latter effect being a mechanical, the former a vital operation. A more minute examination of other organs was not allowed. The heart I have as a wet preparation.

